# Magnesium Sulfate-Induced Anaphylaxis: A Case Report and Review of the Literature

**DOI:** 10.7759/cureus.46071

**Published:** 2023-09-27

**Authors:** Saurab Karki, Mahyar Toofantabrizi, Somika Basnet

**Affiliations:** 1 Department of Internal Medicine, MedStar Franklin Square Medical Center, Baltimore, USA; 2 Department of Internal Medicine, Kist Medical College and Teaching Hospital, Lalitpur, NPL

**Keywords:** case report, hypersensitivity, epinephrine, allergy, anaphylaxis, magnesium sulfate

## Abstract

Anaphylaxis due to magnesium sulfate is uncommon with very few reported cases. We report a case of a 28-year-old female who had low serum magnesium and was given magnesium sulfate. She developed pruritic urticarial rash, hypoxemia, and stridor. Anaphylaxis was diagnosed, and she received epinephrine, diphenhydramine, and oxygen therapy causing resolution. The study also discusses similar published cases and their presentation and treatment. We have briefly overviewed the clinical criteria for the diagnosis of anaphylaxis. The study also intends to make the clinician consider anaphylaxis while administering magnesium sulfate for any reason.

## Introduction

Magnesium sulfate is an agent that has been used widely in obstetrics as a prophylactic drug against seizures in pre-eclampsia and to prevent seizures from recurring in eclampsia [1,2]. It can also be used as a neuroprotective agent in cases of preterm labor [3]. In addition, magnesium sulfate preparation can be used in hypomagnesemia where the oral form is preferred in asymptomatic cases while the intravenous (IV) form is preferred in symptomatic cases [4]. Hypersensitivity reactions against magnesium sulfate are a very rare occurrence. To our knowledge, there are only three reported case reports with hypersensitivity to magnesium sulfate [5,6]. There is another report where the use of magnesium caused nonallergic anaphylaxis [7].

We report a case of a 28-year-old female who developed skin allergies and respiratory symptoms following exposure to magnesium sulfate. The drug was given to correct the electrolyte imbalance. This study also intends to briefly review the previous cases of allergic reactions to magnesium sulfate.

## Case presentation

A 28-year-old female presented to the emergency room with a syncopal episode and chest discomfort. Past medical history was significant for developing an anaphylactic reaction while receiving an iron and magnesium sulfate infusion one year back. The reaction was assumed to be due to iron therapy as there was no significant published literature regarding allergic reaction to magnesium, and the patient also had not undergone a skin allergy test.

The patient was admitted for observation and workup for syncope. The patient had a lower potassium level of 3.3 mmol/L (reference range: 3.4-4.5 mmol/L) and a low-normal magnesium level of 1.8 mg/dL (reference range: 1.6-2.6 mg/dL), so she received 40 mEq of potassium chloride and a 2 g infusion of magnesium sulfate. Thirty minutes later, she began developing pruritic urticarial rashes of the face, arms, neck, and chest, along with flushed skin and shortness of breath. She received IV diphenhydramine and IV famotidine with improvement. Approximately 20 minutes later, the patient had a recurrence of symptoms including diffuse redness, feeling hot, and pruritus of the face, neck, and chest. The patient was tachypneic for up to 40 breaths per minute. Her pulse rate along with her blood pressure was normal. The medical team could also observe stridor and a drop in oxygen saturation to 80% on room air associated with coughing. Intramuscular epinephrine was administered, followed by a second dose of IV diphenhydramine and oxygen therapy, prior to the arrival of the rapid response team. She was transferred to the intensive care unit (ICU) for further management and care. Her ICU stay was relatively normal.

## Discussion

Anaphylaxis is a rapid-onset, potentially life-threatening condition that can lead to death [8]. The National Institute of Allergy and Infectious Diseases/Food Allergy and Anaphylaxis Network (NIAID/FAAN) criteria are a clinical one where the presence of one of the three criteria is sufficient to make a diagnosis [8,9]. The criteria include the following: (1) illness occurring within minutes to several hours that involves the skin, mucosal surfaces, or both (e.g., flushing or pruritus, generalized hives, or swollen tongue with either respiratory compromise {hypoxemia, dyspnea, stridor, wheeze, or reduced peak expiratory flow (PEF)} or decreased blood pressure with associated features of end-organ failure {syncope, incontinence, or hypotonia leading to collapse}); (2) illness that occurs within minutes to several hours after exposure to a likely allergen with two or more of skin-mucosal tissue involvement, respiratory compromise, persistent gastrointestinal symptoms, or decreased blood pressure or its associated symptoms; and (3) decreased blood pressure that occurs within minutes to several hours of exposure to a known allergen. For infants and children, decreased blood pressure is age-specific low systolic pressure or more than 30% decrease from the baseline, while for adults, it is a systolic pressure of less than 90 mmHg or a reduction of more than 30% from one’s baseline.

The use of these criteria has a sensitivity of around 96.7% and a specificity of 82.4% [10]. Epinephrine is the treatment of choice [8].

We searched PubMed and Google Scholar to date and could collect only three published reports. The reports consisted of any cases with the use of magnesium sulfate causing hypersensitivity reactions. In a study by Thorp et al., they reported two cases where magnesium sulfate was used for preterm labor. In the first case, a 32-year-old female with intrauterine pregnancy at 29 weeks of gestation was given an intravenous dose (4 g) of the drug, resulting in an intensely pruritic urticarial rash on her hands, trunk, and face. There was no other system involvement, and treatment consisted of withdrawal of medicine and 50 mg of diphenhydramine following which there was complete resolution. Likewise, in the second case, a 29-year-old female at 34 weeks of gestation had received 4 g of loading dose, while the infusion rate was subsequently maintained at 2 g/hour. She also developed a similar urticarial rash that also extended up to the abdomen. For her, discontinuing magnesium sulfate caused the disappearance of the rash. Both the patients denied similar histories in the past and also denied the offered skin allergy tests [5]. Their case summary is mentioned in Table 1.

**Table 1:** Summary of indications of MgSO4, presentation of allergic reactions following exposure, and their treatment for previously published cases. MgSO_4_: magnesium sulfate; Mg, magnesium

Report year	Author	Age (years)	Sex	Indication of MgSO_4_	Presenting features	Treatment
1989	Thorp et al. [5]	32	Female	Preterm labor	Urticarial pruritic rash	Withdrawal of MgSO_4_ and diphenhydramine
		29	Female	Preterm labor	Urticarial rash	Withdrawal of MgSO_4_
2007	Al-Fares et al. [7]	19	Male	Low serum Mg	Flushing, pruritus, hypotension, tachycardia, and tachypnea	Chlorpheniramine, epinephrine, hydrocortisone, intravenous fluids, and promethazine
2023	Coan et al. [6]	65	Female	Low serum Mg	Swollen tongue, drooling, urticarial rash, and tachypnea	Epinephrine, dexamethasone, famotidine, and diphenhydramine
2023	Our case	28	Female	Low serum Mg	Urticarial rash, tachypnea, coughing, stridor, and hypoxemia	Diphenhydramine, epinephrine, and oxygen therapy

There was a correspondence to the editor by Al-Fares et al., which reported a 19-year-old male who had presented following a motor vehicle accident and had a low normal magnesium level [7]. Magnesium sulfate infusion was used for replacement. The patient developed flushing and pruritus one hour following infusion, which was treated with intramuscular chlorpheniramine. Due to a continuous low level of magnesium, the infusion was restarted on the third day. This time, the patient developed hypotension, tachycardia, and tachypnea in addition to skin findings requiring epinephrine, hydrocortisone, intravenous fluids, and promethazine. However, skin prick testing performed around one year later was non-revealing [7].

In another case report by Coan et al., they presented a case of a 65-year-old female who received two infusions of 2 g of magnesium sulfate. After the second infusion, she was noted to have a swollen tongue, drooling, pruritic urticarial rash throughout the body, and tachypnea [6]. She received epinephrine, dexamethasone, famotidine, and diphenhydramine. The patient was observed in the ICU and had an uneventful recovery. No skin testing was performed [6].

Among the cases, it can be observed that all cases had reactions within an hour after the initiation or completion of the infusion. All cases had skin involvement with a pruritic urticarial rash. Two cases had only skin rash, while the other two cases had respiratory system involvement such as tachypnea, swollen tongue, and drooling. Diagnosis was made clinically in all cases with skin allergy testing performed only in one case, which was negative. Magnesium infusion was stopped, and antihistamines were given as a part of the treatment. Two of the cases also required epinephrine and corticosteroids to alleviate symptoms.

In our case, the patient developed a skin reaction, stridor, and hypoxemia within one hour of infusion. The diagnosis of anaphylaxis was made based on NIAID/FAAN clinical criteria with skin involvement acutely along with respiratory involvement [8,9]. The treatment consisted of diphenhydramine, epinephrine, and oxygen therapy.

## Conclusions

Magnesium sulfate is one of the most frequently used medicines in clinical practice. It can be used in cases of low serum magnesium levels or in obstetric cases with preterm labor. In rare instances, it can be a culprit to cause anaphylactic reactions. So, it is advisable to consider this reaction before giving the medicine. The diagnosis of anaphylaxis is clinical, and the management of such reactions is similar to those caused by other substances.
